# Accuracy of Dynamic Navigation System Workflow for Implant Supported Full Arch Prosthesis: A Case Series

**DOI:** 10.3390/ijerph17145038

**Published:** 2020-07-13

**Authors:** Luigi V. Stefanelli, George A. Mandelaris, Alessio Franchina, Nicola Pranno, Michele Pagliarulo, Francesca Cera, Fabio Maltese, Francesca De Angelis, Stefano Di Carlo

**Affiliations:** 1 Department of Oral and Maxillo-Facial Sciences, Sapienza University of Rome; 00185 Rome, Italy; gigistef@libero.it (L.V.S.); francesca.deangelis@uniroma1.it (F.D.A.); stefano.dicarlo@uniroma1.it (S.D.C.); 2 Private Practice, Periodontics and Dental Implant Surgery, Chicago, IL 60181, USA; gmandelaris@periodontalmedicine.org; 3 Department of Graduate Periodontics, College of Dentistry, University of Illinois, Chicago, IL 60612, USA; 4 Private Practice, Periodontics and Dental Implant Surgery, 36100 Vicenza, Italy; alessiofranchina@icloud.com; 5 Dental Student University of Plovdiv-Bulgary, 4000 Plovdiv, Bulgary; michele.pagliarulo2000@gmail.com (M.P.); francesca.cera0@outlook.it (F.C.); 6 Private Practice, 00192 Rome, Italy; drfabiomaltese@gmail.com

**Keywords:** atrophic maxilla, computer aided implantology, navigation implantology, totally edentulous patients

## Abstract

A minimally invasive implant treatment approach for future full arch implant prosthetic rehabilitations of trophic jaws represents a challenge. An optimal implant planning is strongly related with an accurate merge of the prosthetic information and the radiographic data. To comply with that, most computer aided implantology (CAI) systems require additional steps, as radiographic stents or fiducial markers to overlap digital jaw scans to cone beam computed tomography (CBCT) data. Using dynamic CAI, residual teeth (up to three) make it possible for the merge to avoid new radiographic scans. An additional challenge is the treatment involving immediate implants compared with delayed implants placed into healed bone. As for other static CAI systems, the operator’s experience and the quality of the CBCT data make the planning affordable and secure the entire implants placement procedure. The literature reports accuracies in terms of comparison between placed implants and planned implants, following a double CBCT approach, based on radiographic volume overlapping. Thirteen consecutive future totally edentulous patients (77 implants), divided into two groups (group A: 3–4 teeth traced; group B: 5–6 teeth traced) requiring a full arch implant prosthetic rehabilitation were included in the reported case series. A dynamic CAI was used to plan and to place all implants following all the recommended digital steps. The software used provided a tool (Trace and Place) that made the merge between X-ray views of the residual teeth and their own positions possible. This method definitely registered that teeth positions comply with the required accuracy live check. After implants placement, a post-operative CBCT was taken in order to evaluate the deviations of the achieved implants at coronal, apical, and depth level as well as angular deviations. Statistically significant radiological mean difference between the two groups was found in the coronal position of implants (0.26 mm, *p* < 0.001), in the apical position of implants (0.29 mm, *p* < 0.001), in the depth of implants (0.16 mm, *p* = 0.022), and in the angular deviation (0.7, *p* = 0.004). The use of the TaP technology for the treatment of the patients with at least three stable teeth that need to be removed for a totally implant prosthetic treatment is a promising technique. The performed accuracy analysis demonstrated that this digital protocol can be used without a loss of accuracy of the achieved implants compared to planned ones.

## 1. Introduction

A minimally invasive implant treatment approach for the full arch implant prosthetic rehabilitation of atrophic jaws represents a challenge [1]. In these cases, an insufficient bone volume in the posterior area of the jaws to be treated could be solved by using short or tilted implants as an alternative to bone graft [2,3]. If an immediate loading is required, the use of tilted longer implants instead of short implants allows higher torque values to be reached [4]. A minimally invasive approach and immediate loading decreases treatment time, morbidity (avoidance of a second surgery to expose the implant), and the requirement of a mobile denture with its related functional and aesthetic disturbance [5,6,7]. The literature reports that the success rates in the short and medium term of immediate implants are high (90–100%) [8]. An additional challenge is represented for those cases where immediate implants are planned—the placement of an implant in a post extracted socket compared to a healed ridge seem to be more difficult in order to obtain the needed primary stability [9] The insertion of tilted implants parallel to the anterior sinus wall with a minimally invasive approach (i.e., flapless) requires the use of a computer-aided implantology (CAI) [10,11]. Two different approaches of CAI can be used—static guides or dynamic navigation systems [12,13,14].

Static computer-aided implantology is based on the use of a surgical guide, which can be printed using a stereolithographic printer or drilled using a numeric controlled machine. The term static indicates that the operator plans the position of the implants in software and then this direction is replicated in a surgical guide. In this way, the operator cannot change the planned position of the implants during the surgery. The term dynamic indicates that it is possible to change the position of the implants during surgery because the use of dynamic technologies allows the user to track the CT or the cone beam computed tomography (CBCT) of the patient in real time, and the osteotomy is done free-hand following the position of the drill on a monitor [13,14].

Even if both methods report good values of accuracy [14,15], when the patient to be treated has some residual teeth to be removed, the use of CAI can be an additional obstacle [16]. In any cases, in fact, both methods require a radiological marker, so that if the patient has a recent CBCT a new one is needed to allow the clinician to follow the CAI method.

A novel approach is possible by means of using a new workflow of a dynamic navigation system called Trace and Place (TaP) technology that uses an existing CBCT (for patients who have at least three remaining teeth) rather than preparing a custom stent and scanning the patient two times. Instead, the registration of the jaw with its computer tomography (CT) image is accomplished by tracing the existing teeth as natural fiducial markers [17,18] and performing the guided surgery without any intraoral appliance. This provides a fully digital implant—the prosthetic treatment workflow [19,20].

This approach is valuable if compared with other dynamic CAI systems because it does not need a radiological stent or radiopaque markers. 

The primary aim of this study was to assess if there was any accuracy difference when a wider area of overlapping between the CBCT and the arch was traced (3–4 teeth traced vs. 5–6 teeth traced).

H_0_: There is no difference in the accuracy of 3–4 teeth traced versus 5–6 teeth traced compared to planned implants.

The secondary aim of this study was to evaluate the accuracy of this workflow in terms of deviation values between the planned and achieved implants.

## 2. Materials and Methods

### 2.1. Methods

#### Study Design

This study was a case series of 13 consecutive implant treatments of future fully edentulous patients. This study protocol was approved by the Department of Oral and Maxillofacial Sciences—Sapienza, University of Rome. The outcomes were evaluated both clinically and radiographically after three months post-loading.

### 2.2. Study Population/Demographics

Thirteen partially edentulous patients were consecutively scheduled between 1 February 2018 and 30 November 2018 for a full arch implant rehabilitation at the Department of Periodontics and Implant Dentistry at the Policlinico Umberto I, Sapienza University of Rome, Italy.

#### 2.2.1. Inclusion Criteria

(1) Arches partially edentulous that need a full arch implant prosthetic rehabilitation; 

(2) Patients with at least three stable/non-mobile teeth;

(3) Eighteen years old or older

#### 2.2.2. Exclusion Criteria

(1) Patients with general contraindications to implant surgery;

(2) Patients with systemic diseases that could influence the outcome of the therapy (i.e., diabetes with HbA1c ≥ 6.5%, osteoporosis or use of bisphosphonate medications);

(3) Patients with a history of radiation to the head and neck region;

(4) Patients who are pregnant or nursing.

A written informed consent was obtained from each patient after a detailed description of the proposed surgical and prosthetic study protocol and treatment were given. The protocol was in accordance with the 1975 Declaration of Helsinki on medical protocols and ethics and its later amendments. The clinical study protocol included the use of a post-operative CBCT scan to assess the accuracy of achieved implants compared to those planned and the position and angulation of the implants relative to the virtual plan. This study protocol was approved by the Department of Oral and Maxillofacial Sciences—Sapienza, University of Rome (protocol identifying number: 582/17).

### 2.3. Trace and Place (TaP) Protocol

The workflow has been described in previous publications [17,18]; never the less, it is described here briefly.

The TaP protocol consists of the following three steps: (1) Plan—The creation of a virtual surgical plan on the basis of the volumetric DICOM (Digital Imaging Communication in Medicine) data acquired from a CBCT scan. (2) Trace—The registration of the patient’s jaw to their CBCT. This is done by tracing high contrast landmarks selected/marked on the CBCT. (3) Place—The implant placement is navigated according to the plan.

### 2.4. Plan

A cone beam computed tomography CBCT (Scanora3Dx, Soredex, Tuusula, Finland) and an intraoral surface scan IOS (Medit i500, Medit Corp., Seoul, South Korea) of each patient were taken. An ideal virtual wax-up of the teeth to be replaced was completed. Both the Digital Imaging and Communications in Medicine (DICOM) files (from the CBCT) and the stereolithography (STL) files (from the IOS) were merged into the Navident software (ClaroNav Inc., Toronto, ON, Canada) and overlapped semi-automatically to the residual teeth using the provided mesh-to-image registration tool.

**Figure 1:**
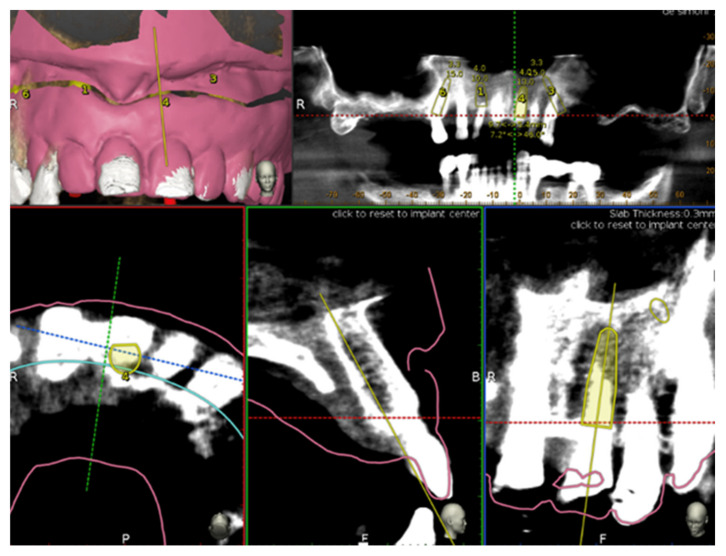
Implant planning by using stereolithography (STL) file as reference for a prosthetic driven implantation.

Implants placement was then prosthetically planned utilizing the digital wax up of the missing teeth (Figure 1). As part of the planning, the surgeon selected 3–6 landmarks on hard tissue structures, typically teeth, to be used as the starting points for tracing.

### 2.5. Trace

**Figure 2:**
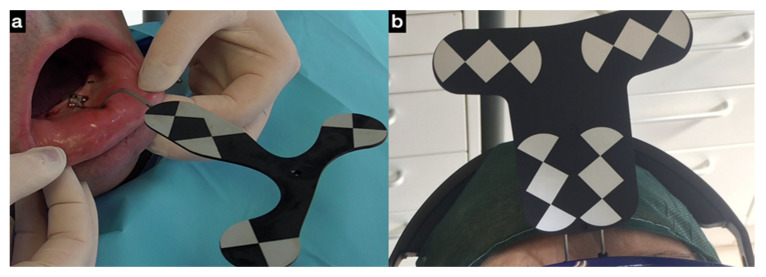
(**a**)The jaw tracker anchored with three bone screws to the lower jaw where the surgery is planned; (**b**) the head tracker is alternatively used to track the maxillary jaw.

In order to track the patient’s jaw, an optical tracking tag needs to be affixed to the jaw on which the surgery has to be performed. This requires a JawTracker (ClaroNav Inc., Toronto, ON, Canada) (a combination of the optical tag and a bendable metal wire) to be connected to 1–2 teeth in the residual dentition with a light-cured composite resin (Figure 2a). Alternatively, and only in the maxilla, a HeadTracker (ClaroNav Inc., Toronto, ON, Canada) can be used to track the maxilla by placing it directly on the patient’s head (Figure 2b). Then, tracing can be performed starting at the landmark locations. During tracing, the surgeon slides the tracer’s ball tip in full contact over the surface of each landmark until a 15 cm path has been traced.

To address the secondary study aim, two groups were randomly created in order to assess if a 3–4 teeth tracing (group A) performed equally well compared to a 5–6 teeth tracing (group B).

**Figure 3:**
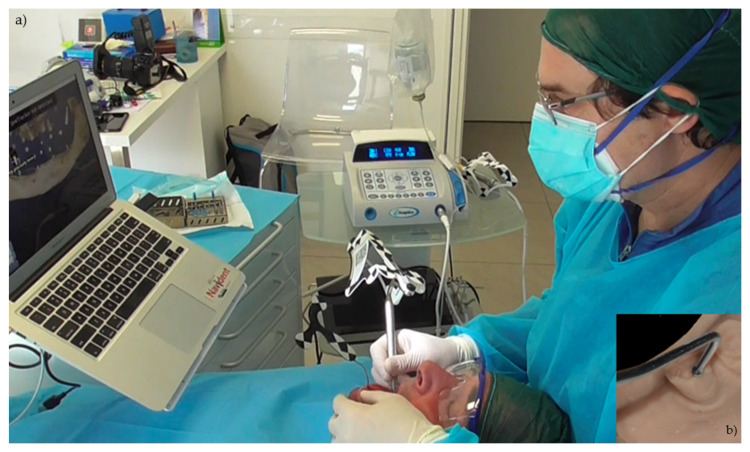
The picture shows the surgeon and the laptop screen where the tracing process is visible (**a**); in the right corner (**b**) the ball tip of the tracer.

**Figure 4:**
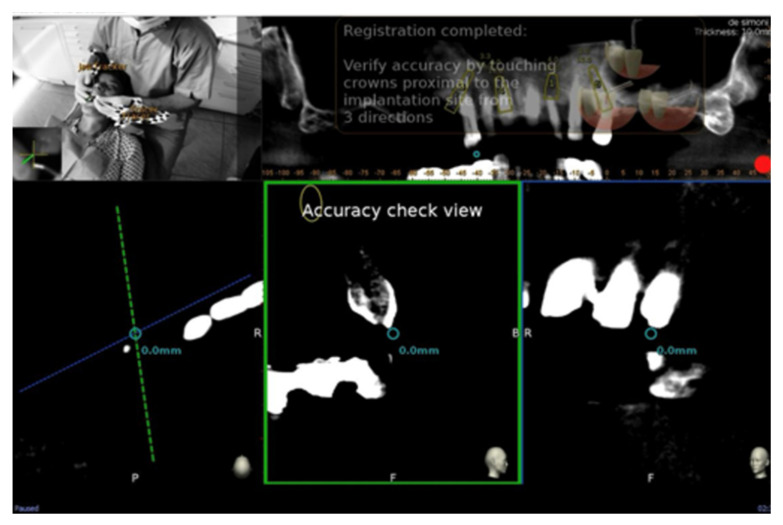
The surgeon can then verify the registration accuracy by touching with the tracer’s ball tip on the patient’s landmark from several aspects and comparing the physical location of the tip with its on-screen representation on the system’s screen.

After tracing all selected landmarks/teeth, the software automatically performs the registration process. The sample traced points are aligned with strong edges in the CBCT image. The complete trace and registration process takes 1–2 min on average. The accuracy of the trace registration can be evaluated instantly in the software by touching the patient’s teeth with the tracer’s ball tip from the buccal, lingual, incisal/occlusal and proximal planes and comparing the actual physical location of the tracer tip with its own on-screen representation on the system’s display (Figure 3 and Figure 4). The same check can also be carried out in edentulous cases by touching bone screws intentionally placed before taking CBCT scan. If the registration accuracy is not satisfying, the tracing step can be immediately repeated.

### 2.6. Place

**Figure 5:**
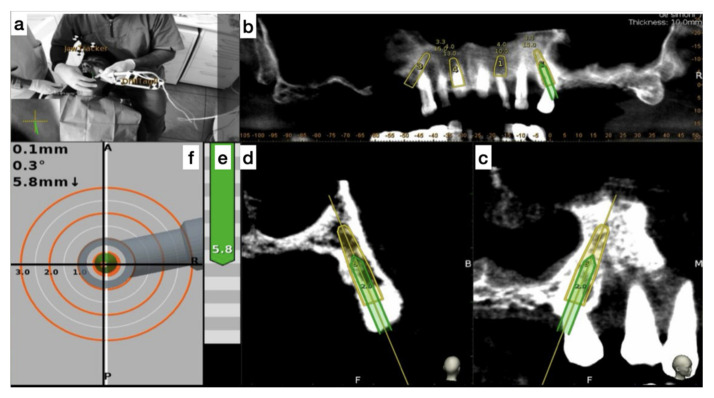
The figure indicates the several views on the screen during surgery and the advancing of the drill into the bone. (**a**) Tracker video stream, (**b**) panoramic view, (**c**) mesiodistal section view, (**d**) Bucco-lingual section view, (**e**) depth indicator, and (**f**) target view.

The handpiece drill axis and drill tip are calibrated, and a second verification of the accuracy is carried out in the same manner as the tracer. Once the drill axis and drill tip accuracy are verified, the navigated implant placement can be carried out following the target view, which allows the clinician to verify, in real time, the entry point, depth, and angulation of the planned osteotomy as related to the plan. The other views that the clinician can see on the screen enable him/her to follow the position of the drill during the osteotomy in the coronal and sagittal views (Figure 5).

### 2.7. Surgical Treatment

Before the surgical treatment, all patients underwent an oral hygiene protocol that consisted of polishing when needed, and supra- and subgingival debridement. One hour prior to surgery, patients received prophylactic antibiotic therapy with 2 g of Amoxicillin (Augmentin, GlaxoSmithKline, London, UK). Immediately before the procedure, they were instructed to rinse with a 0.2% chlorhexidine digluconate solution (Corsodyl, GlaxoSmithKline Consumer Healthcare, Genval, Belgium) for 2 min. All surgical procedures were performed by the same experienced surgeon (L.V.S.). Local anesthesia with 2% mepivacaine 1:100,000 adrenalin (Carbocaine, AstraZeneca, Milan, Italy) was used.

The surgical strategy first provided the placement of implants (TPA, AZ implant, San Lazzaro di Savena, Bologna, Italy) at healed sites and then the extractions of the teeth where immediate implants were planned and at the end the others remaining extractions.

**Figure 6:**
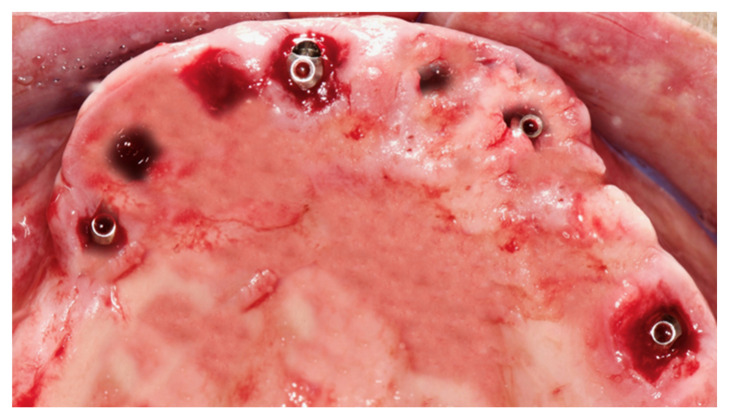
Multi-unit abutment were screwed on the implants after the surgery.

After implant insertion and teeth extraction, the multi-unit abutments were screwed (Figure 6), and an impression was taken to prepare a provisional screwed retained prosthesis.

**Figure 7:**
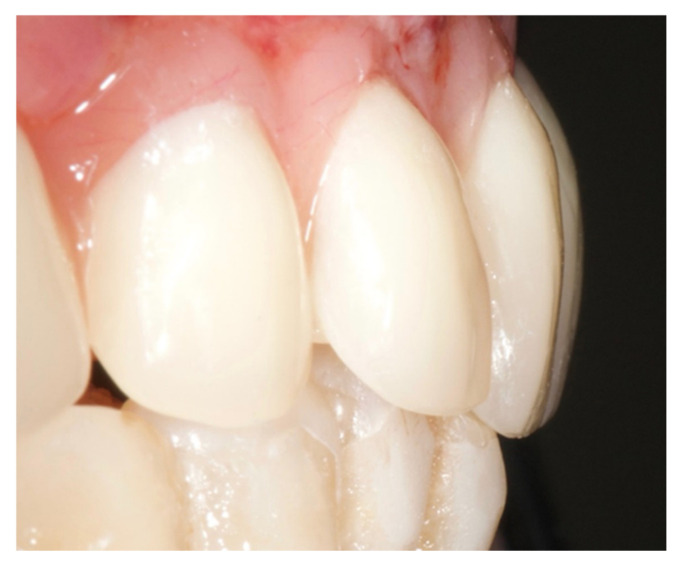
Provisional prosthesis was screwed, and the occlusal check was done.

The healing screws were screwed after 6 h, the provisional prosthesis was screwed, and an occlusal check was performed (Figure 7).

### 2.8. Post-Surgical Protocol

All patients were prescribed amoxicillin (Augmentin, GlaxoSmithKline, London, UK) 1 g twice daily for seven days. After surgery, analgesia was achieved with 200 mg of ketoprofen (Ibifen, Aprilia, Latina, Italy) for a maximum of three times days according to the needs of individual patients. Each patient was instructed to rinse with 0.12% chlorhexidine digluconate (Corsodyl, GlaxoSmithKline Consumer Healthcare S.p.A., Baranzate, Milan, Italy) three times daily for two weeks, to follow a soft diet for one week, and to gently clean with a soft toothbrush while avoiding flossing in the surgical area for the first month post-operatively.

### 2.9. Outcome Measures

#### Post-Surgery Complications

The following post-surgery complications were recorded: (1) lack of primary stability of the implants, (2) post-operative hemorrhage, (3) post-operative infection, and (4) early implant failure.

### 2.10. Accuracy Evaluation Assessment

Each patient had a CBCT before and after implant placement. Two independently calibrated investigators (S.D.C., P.N.) with 10 years of experience in CAI, who were blinded to other aspects of the study, measured the accuracy between the planned and inserted implants. Any disagreement was solved by consensus, and a third investigator was consulted when it was not initially possible to achieve a complete agreement (defined as the difference between the measurements made by the two experts of >0.1 mm). All the investigators had the same skill and level of experience in to use of both Navident software and Evalunav software.

**Figure 8:**
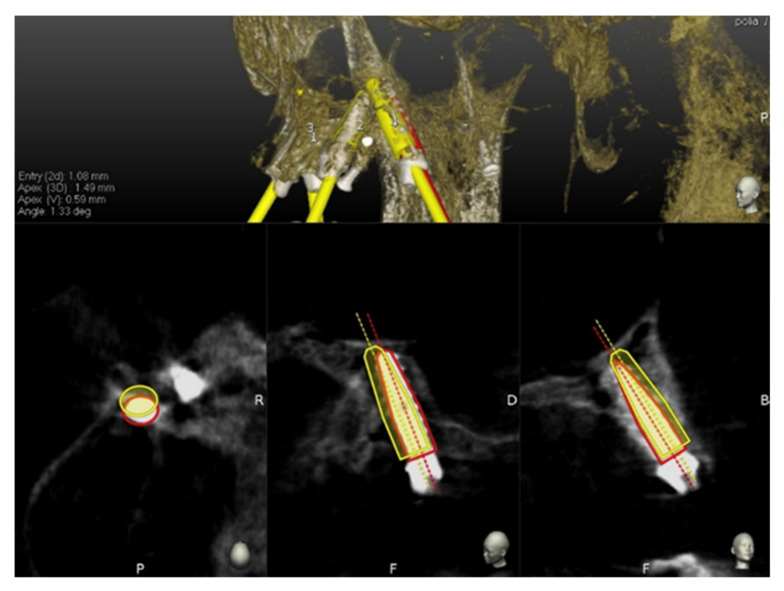
The software automatically fits a model of the implant to its appearance in the post-operative image and computes the angular axis corrected between the planned and actual implant locations (implant inserted with dynamic guidance).

The preoperative surgical plan and the postoperative CBCT were superimposed using an accuracy evaluation application (EvaluNav) provided through the Navident navigation system (Claronav Inc., Toronto, ON, Canada). The registration was done directly between the two volumetric images. The software provides various visualization tools which confirm the two images are precisely aligned and improve the accuracy if the alignment is less than ideal. Once the user is satisfied with the volumetric registration, the software automatically fits a model of the implant to its appearance in the post-operative image and computes the angular axis corrected between the planned and actual implant locations (Figure 8).

### 2.11. Statistical Analysis

The required sample size was calculated using statistics software (GPower 3.1.9.2, Heinrich-Heine-Universität, Düsseldorf, Germany). A power analysis using the independent samples t-test, an alpha level of 0.05, and a medium effect size (f = 0.86) showed that 72 implants would be adequate to obtain 95% power in detecting a statistical difference in the coronal position of implants using 3–4 teeth traced vs. 5–6 teeth traced, assuming a loss to follow-up of 20%.

A database was created using Excel (Microsoft, Redmond, WA, USA). Descriptive statistics including mean ± SD values were calculated for each variable, and box plots were used to evaluate data outliers. The Shapiro–Wilk test was used to determine whether or not the data conformed to a normal distribution.

The independent samples t-test was used to identify statistically significant differences in the coronal position of implants, the apical position of implants, depth of implants and angle of implants between two groups, one in which was used 3–4 teeth traced versus 5–6 teeth traced compared to the planned implants.

Data were evaluated using standard statistical analysis software (version 20.0, Statistical Package for the Social Sciences, IBM Corporation, Armonk, NY, USA). In each test, the cut-off for statistical significance was *p* ≤ 0.05.

## 3. Results

A total of 13 patients (77 implants) were enrolled in the study (seven males, six females; age 68.15 ± 9.22 years). The patients were distributed in the two groups—group A (33 implants), in which 3–4 teeth were traced, and group B (44 implants) in which 5–6 teeth were traced.

Post-operative complications were not reported by the 13 patients. Seventy-three of 77 implants (94.8 % of integration ratio) were osseointegrated after four months of healing (four implants were lost during the period of healing). These early implant failures represented the only four complications reported in the 13 patients treated.

**Figure 9:**
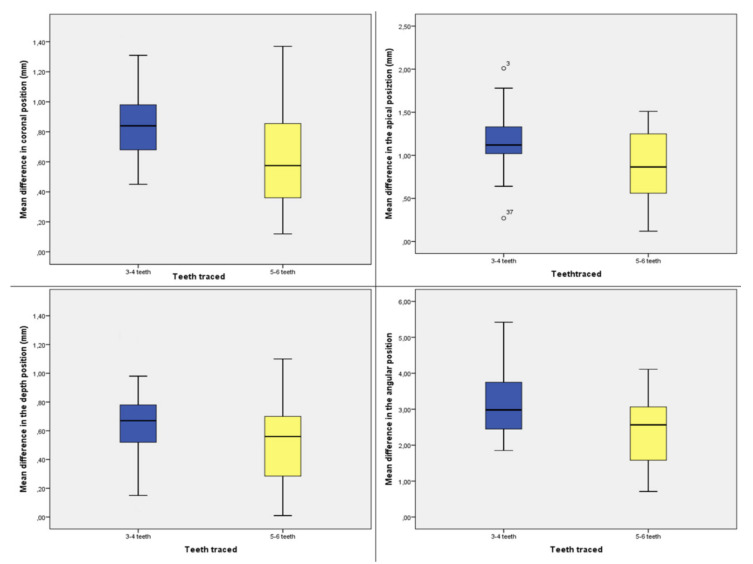
Box plots showing the median, quartile, and minimum and maximum values of the mean differences between the planned implants and inserted implants in patients with 3–4 or 5–6 teeth left in the coronal position of implants (mm), the apical position of implants (mm), depth of implants (mm), and angle of implants.

The mean differences in the coronal position of implants, the apical position of implants, depth of implants, and angle of implants between the two groups compared to the planned implants are reported in Figure 9.

Only two outliers were detected, and the assumption of normality, assessed by the Shapiro–Wilk test, was not violated. The independent samples t-test showed a statistically significant mean difference between the two groups in the coronal position of implants (3–4 teeth group: 0.720 ± 0.322 mm; 5–4 group: 0.61 ± 0.328 mm; *p* < 0.001), the apical position of implants (3–4 teeth group: 1.168 ± 0.313 mm; 5–4 group: 0.877 ± 0.370 mm; *p* < 0.001), depth of implants (3–4 teeth group: 0.658 ± 0.297 mm; 5–4 group: 0.501 ± 0.280 mm; *p* = 0.022), and angle of implants (3–4 teeth group: 3.103 ± 1.019°; 5–4 group: 2.407 ± 0.983°; *p* =0.004) (Table 1).

## 4. Discussion

This study evaluated the accuracy of a Digital Dynamic Navigation System workflow in planning and inserting dental implants for transitioning the terminal natural dentition to an implant-supported full arch prosthesis (arches with at least three stable teeth before the surgery). 

A full rehabilitation of both arches is always a challenge because optimal implant planning is strongly related with an accurate merge of the prosthetic and the radiographic data. When the patient is totally edentulous, a new CBCT of the patient with a wax up in which some radiological points of reference are added is required in order to overlap the STL of the wax up to the CBCT. On the other hand, the patients that have some residual teeth to be removed before the full implant prosthetic treatment represent a great opportunity because the prosthetic information could be overlapped to the residual teeth avoiding the CBCT of the patient wearing the wax up.

Another challenge of full implant prosthetic rehabilitation is the limited residual bone of the posterior areas. In these cases, tilted implants are used to laterally bypass any anatomical limitation, such as the maxillary sinus in the upper arch and the inferior alveolar nerve in the lower arch, and to use most of the residual bone for a better anchorage. Malò et al. [21,22] demonstrated that the use of two posterior tilted implants and two frontal implants are efficient in treating these patients.

For the above-mentioned reasons, the accuracy of implant insertion in the full implant prosthetic restorations is important not only for avoiding damages to the anatomical structures but also to have a predictable result in accordance to the planning and its related aesthetic outcomes. The use of static or dynamic CAI is strongly recommended because both methods are more accurate than free-hand.

Shen et al. [11] inserted 52 implants using only the preoperative planning without surgical templates and 57 implants with surgical templates and reported the deviations between the two groups. Variation at the implant shoulder of the group without surgical templates was 1.18 ± 0.72 mm, apex 1.43 ± 0.74 mm, angulation 4.21 ± 1.91 mm, and depth 0.54 ± 0.29 mm, whereas the variation in group with the use of surgical template was 2.07 ± 0.51 mm (*p* < 0.01), 2.89 ± 1.02 mm (*p* < 0.01), 8.84 ± 4.64 mm (*p* < 0.05), and 0.78 ± 0.33 mm (*p* > 0.05). They concluded that the use of surgical guide templates can achieve higher precision and accuracy in implant shoulder, apex, and angulation, which is much more suitable for complicated procedures and conditions such as the flapless approach, immediate loading, aesthetic restoration, and insufficient bone height. Vercruyssen et al. [23] reported the accuracy by comparing the insertion of the implants of 72 fully edentulous jaws using pilot-drill templates (static guides) or free-hand (mental navigation). They reported an error of 1.4 (0.7) mm at the entry point, 1.6 (0.7) mm at the apex and 3.0 (2.0) degrees from the angular standpoint by using static guides and 2.8 (1.5) mm at entry point, and 2.9 (1.5) mm at the apex and 9.9 (6.0)° for angle deviations by using free-hand. With this kind of deviation (i.e., 3 mm at apex and more than nine degrees as angular error), attempting a free hand approach could represent a high risk for the anatomical structures, especially in the tilted implants.

Block et al. [24] treated 100 patients in a multicenter study (three surgeons) using dynamic navigation (X-Guide, X-Nav Technologies). They reported the following data of accuracy: 0.87 (0.42) mm at the entry point (lateral/2D), 1.56 (0.69) mm at the apex (3D) and 3.62° (2.73°) for angle deviations by using dynamic navigation while the mean deviations for non-guided surgeries at the entry point, the apex point and the angular discrepancies were, respectively, 1.15 (0.59) mm, 2.51 (0.86) mm, and 7.69° (4.92°).

Block et al. [25] reported also the accuracy of 714 implants inserted in 478 patients by using a dynamic navigation system vs. free-hand. The mean deviation at entry point was 1.16 mm, at the apical point was 1.29 mm, and the mean angle discrepancy was 2.97° using a dynamic navigation system and 1.78 mm at the entry point, 2.27 mm at the apical point, and 6.5 degrees as angular discrepancy when the implants were inserted free-hand.

Aydemir and Arisan [26] in a split mouth study (navigation vs. free-hand) inserted 92 implants in 32 patients with bilateral edentulism in the posterior maxilla (Kennedy class I). The deviations reported were 1.70 mm at entry point, 2.51 mm at apex, and 10.04 degrees as angular deviation by using free-hand; 1.01 mm at entry point, 1.83 mm at apex, and 5.59 degrees as angular deviation by using dynamic navigation system.

Stefanelli et al. [18] reported in a retrospective observational study on 231 implants (89 arches) an error of 0.71 mm at the entry point, 1 mm at the apex, and a mean angular error of 2.26 degrees.

Pellegrino et al. [27] reported by inserting 10 implants with a flapless approach by using a dynamic navigation system a mean deviation of 1.10 mm at coronal point, 1.27 mm at apical point, 0.49 at depth, and 5.28 degrees. It was also reported that the insertion of 50 implants was necessary to bed in the learning curve.

In any case, both methods need a radiological stent before scanning the patient, so if a recent CBCT was done without the use of radiological stent, a new one is necessary. In particular, when a partially edentulous patient needing a full implant prosthetic rehabilitation is treated with surgical guides, two surgical templates are often used; in fact, the first is used to create the anchor pin sites before the teeth extractions, and the second is used to perform implant surgeries by the surgical guide that is made stable by some pins (3–4) anchored to those bone holes prepared with the previous guide. In this way, a correlation between the CT/CBCT scan and the reality is maintained. The use of radiological guides/stents and single or multiple surgical templates represents a cost both for dentist and patient, increases the time of the procedure, and adds a source of error in the CAI workflow.

The proposed method in this article is based on Trace and Place technology, i.e., the use of the residual teeth (at least three stable teeth) as natural radiological marker.

The primary aim of this study was to evaluate if this technique was as accurate as the ones already used as static CAI. There is only one study in the literature about this method, written by Stefanelli et al. [28], and it regards partially edentulous patients. They treated 59 partially edentulous arches for a total of 136 implants placed and reported an accuracy of 0.67 mm at the coronal level, 0.9 mm at the apical level, 0.55 mm in depth, and 2.5 degrees as angular error.

In this study the reported discrepancies of the insertion of 77 implants were 0.72 mm at coronal level, 1.00 mm at apical level, 0.56 mm at depth, and 2.7° as angular deviation. These results are comparable with the ones reported by using the same TaP technology in the partially edentulous patients and other methods of CAI, but the advantages of this method are multiple—no radiological stent and surgical guides (for static CAI)/ mini-implant (for dynamic systems) are required, the clinician could use the CBCT of the patient (also if both arches need to be treated) if already available, no devices have to be placed in the mouth during the surgery, and a full digital implant-prosthetic planning and surgery are possible.

Other advantages of the dynamic navigation systems over static guides include that dynamic guidance allows the surgeon to visualize implant site development when drills are in function; that deviation from the predetermined plan can be seen in “real time” and changes can be made at time of surgery; that dynamic navigation allows for direct visualization of the surgical field at all time; that dynamic navigation can be used on patients with limited opening mouth and in the posterior area of the mouth such as the second molar sites; tight single-tooth situations can be fully guided using dynamic guidance, as the dynamic guide has not restricted by drill tube size (i.e., in the anterior mandibular incisor sites); that implant size is not limited with dynamically guided systems, as they are with static guides; dynamic navigation systems are completely open and do not require special instrumentation; and that CBCT, planning, and surgery can be done in a single day.

The secondary aim of this study was to evaluate if there was any difference in terms of accuracy when 5–6 teeth instead 3–4 teeth were achieved for tracing. It resulted that when 5–6 teeth instead 3–4 teeth were used for the tracing, a statistically significative difference was found in all analyzed deviations.

This result was due to the wider area of the arch, including more teeth to be traced and overlapped. To understand this point is very important because less deviations have important implications for patient safety and surgical success, especially when tilted implants are used to bypass laterally any anatomical limitation, such as the maxillary sinus in the upper arch and the inferior alveolar nerve in the lower arch

Further research efforts are needed to understand if the position of the residual stable teeth to be removed can affect or influence the present method when the same number of traced teeth is used.

It was reported that 73 of 77 implants (94.8 % of integration ratio) were osseointegrated after four months of healing (four implants were lost during the healing period). Three lost implants belonged to the Group A (3–4 teeth traced), and one belonged to the Group B (5–6 teeth traced).

The use of Trace and Place protocol in the fully edentulous arch is currently experimental and it requires the presence or addition of fixed reference points, such as mini-screws, to ensure sufficient accuracy.

The limitation of this study is that the results referred to a single surgeon in a single practice. Caution should be exercised when interpreting these results on a broader context and generalizing such results among surgeons. Additional similar in-vivo accuracy studies should be undertaken to validate our results so that more data will be available on a broader context in such a challenging yet important rehabilitations.

Another limitation of this study is the limited follow-up of both the inserted implants and the full arch prosthesis.

## 5. Conclusions

The use of the TaP technology for the treatment of the patients with at least three stable teeth that need to be removed for a totally implant prosthetic treatment is a promising technique because it allows the clinician to perform a highly accurate and precise implant placement, even in the case of a close relation between implant path and anatomical structures, often occurring when a totally edentulous patient is treated. Furthermore, using 5–6 teeth traced a statistically significant augmentation of the accuracy in the coronal, apical, in the depth, and in the angular position of the implants compared with the planned ones was found. However, the clinical relevance of this difference is probably negligible.

## Figures and Tables

**Table 1:** Mean difference in the coronal position of implants, the apical position of implants, depth of implants, and angle of implants between the two groups.

Independent Samples Test						
	T-Test for Equality of Means					
	T	Sig. (2-Tailed)	Mean Difference	Std. Error Difference	95% Confidence Interval of the Difference	
					Lower	Upper
Coronal	3.863	0.000	0.264	0.068	0.128	0.400
Apical	3.640	0.000	0.291	0.080	0.132	0.450
Depth	2.344	0.022	0.155	0.066	0.023	0.287
Angular	3.030	0.003	0.697	0.230	0.239	1.155

Independent samples T-test showed a statistical significant difference using 3–4 teeth traced vs. 5–6 traced for all dependent variables.
